# A Cas12a-based CRISPR interference system for multigene regulation in mycobacteria

**DOI:** 10.1016/j.jbc.2021.100990

**Published:** 2021-07-21

**Authors:** Neil Fleck, Christoph Grundner

**Affiliations:** 1 Center for Global Infectious Disease Research, Seattle Children's Research Institute, Seattle, Washington, USA; 2 Department of Pediatrics, University of Washington, Seattle, Washington, USA; 3 Department of Global Health, University of Washington, Seattle, Washington, USA

**Keywords:** CRISPRi, *Mycobacterium tuberculosis*, mycobacteria, Cas12a, gene regulation, Cas, CRISPR-associated protein, CRISPR, clustered regularly interspaced short palindromic repeat, CRISPRi, CRISPR interference, *Msm*, *Mycobacterium smegmatis*, sgRNA, single guide RNA

## Abstract

Mycobacteria are responsible for a heavy global disease burden, but their relative genetic intractability has long frustrated research efforts. The introduction of clustered regularly interspaced short palindromic repeats (CRISPR) interference (CRISPRi) has made gene repression in mycobacteria much more efficient, but limitations of the prototypical Cas9-based platform, for example, in multigene regulation, remain. Here, we introduce an alternative CRISPRi platform for mycobacteria that is based on the minimal type V Cas12a enzyme in combination with synthetic CRISPR arrays. This system is simple, tunable, reversible, can efficiently regulate essential genes and multiple genes simultaneously, and works as efficiently in infected macrophages as it does *in vitro*. Together, Cas12a-based CRISPRi provides a facile tool to probe higher-order genetic interactions in mycobacteria including *Mycobacterium tuberculosis* (*Mtb*), which will enable the development of synthetically lethal drug targets and the study of genes conditionally essential during infection.

The adaptive bacterial immune systems based on clustered regularly interspaced short palindromic repeats (CRISPR) and CRISPR-associated proteins (Cas) have transformed genetic manipulation, and the ease with which they can be programmed has led to their wide use in gene editing in eukaryotes and prokaryotes (1). One application of the CRISPR/Cas system, CRISPR interference (CRISPRi), introduced a new way of gene regulation by coexpressing an inactive Cas9 nuclease with an engineered single guide RNA (sgRNA) that directs the inactive nuclease to a target gene where it blocks transcription rather than cleaves the DNA (2). The prototypical CRISPRi system is based on an inactive type 2-II Cas9 nuclease (dCas9) and has recently also been adapted for use in mycobacteria including *Mtb* (3, 4, 5), for which genetic manipulation has long been an experimental bottleneck.

Genetic manipulation of *Mtb* poses many challenges. The slow growth of *Mtb* makes all manipulations requiring chromosomal changes time-consuming, and while the high rate of illegitimate recombination in *Mtb* has been overcome by expressing heterologous recombineering enzymes (6), allelic exchange and possibly removal of selection markers can still take weeks to months. Tunable gene repression and repression of essential genes before the introduction of CRISPRi required the introduction of regulatable promoters such as the tetracycline- or pristinamycin-inducible promoters (7) or sequences for regulated protein degradation (8) in the chromosomal copy of a target gene. These approaches had varying efficiency, and leaky expression or repression could lead to incomplete control of target genes. While these challenges already complicated the manipulation of single genes, they multiplied for the manipulation of multiple genes at once. As a result, our understanding of genetic interactions in *Mtb* is lagging behind that in other bacteria. For genome-wide studies in *Mtb*, transposon mutagenesis has become a powerful tool, but insertion of transposons produces libraries with undefined mutations that require sequencing for deconvolution. Also, transposon libraries do not include essential genes, the genes that are arguably the most relevant, for example, in drug discovery. CRISPRi genome-wide libraries have been developed for eukaryotes and have been used, for example, for probing the host genetic factors for *Mtb* infection (9). With their defined nature, tunability, and inclusion of essential genes, CRISPRi *Mtb* mutant libraries seem poised for global approaches to understand *Mtb* gene function.

Despite the advantages of CRISPRi, however, knockdown efficiency can vary widely for different target genes, and simultaneous manipulation of more than one gene remains challenging. These limitations have hampered attempts to probe redundant genes, gene families, and higher-order genetic interactions. Natural CRISPR systems are inherently multigene regulatory systems that can principally also be reprogrammed to regulate multiple genes at once. One current limitation of the Cas9-based system, however, is the relatively large size of the sgRNA which requires a crRNA and a tracrRNA that are typically fused to produce the >100 bp long sgRNA. Expression of multiple sgRNAs for multigene knockdown requires stepwise cloning of large individual transcriptional units for each sgRNA and in mycobacteria has shown mostly moderate knockdown efficiency of 2- to 3-fold for most genes (10). The natural diversity of CRISPR systems, however, may offer simpler solutions to CRISPRi in *Mtb*: Recently, the minimal type 2-V CRISPR enzyme Cas12a (previously Cpf1) has been described (11). Cas12a does not require a tracrRNA and combines pre-crRNA processing and interference functions in one enzyme (12). The combination of these biochemical functions makes Cas12a a stand-alone enzyme that at least in some bacteria only requires a synthetic CRISPR array for the generation of mature crRNAs. This simplified system has been rapidly adapted for multiplex gene editing in mammalian cells (13), plants (14), and bacteria (15) and has recently also been exploited for CRISPRi in *Streptomyces* (15), *E. coli* (16), and the cyanobacterium *Synechococcus* (17, 18) (Fig. 1). Here, we adapted this system for CRISPRi in mycobacteria, creating a simple, highly tunable, reversible, multigene regulation platform.

**Figure 1:**
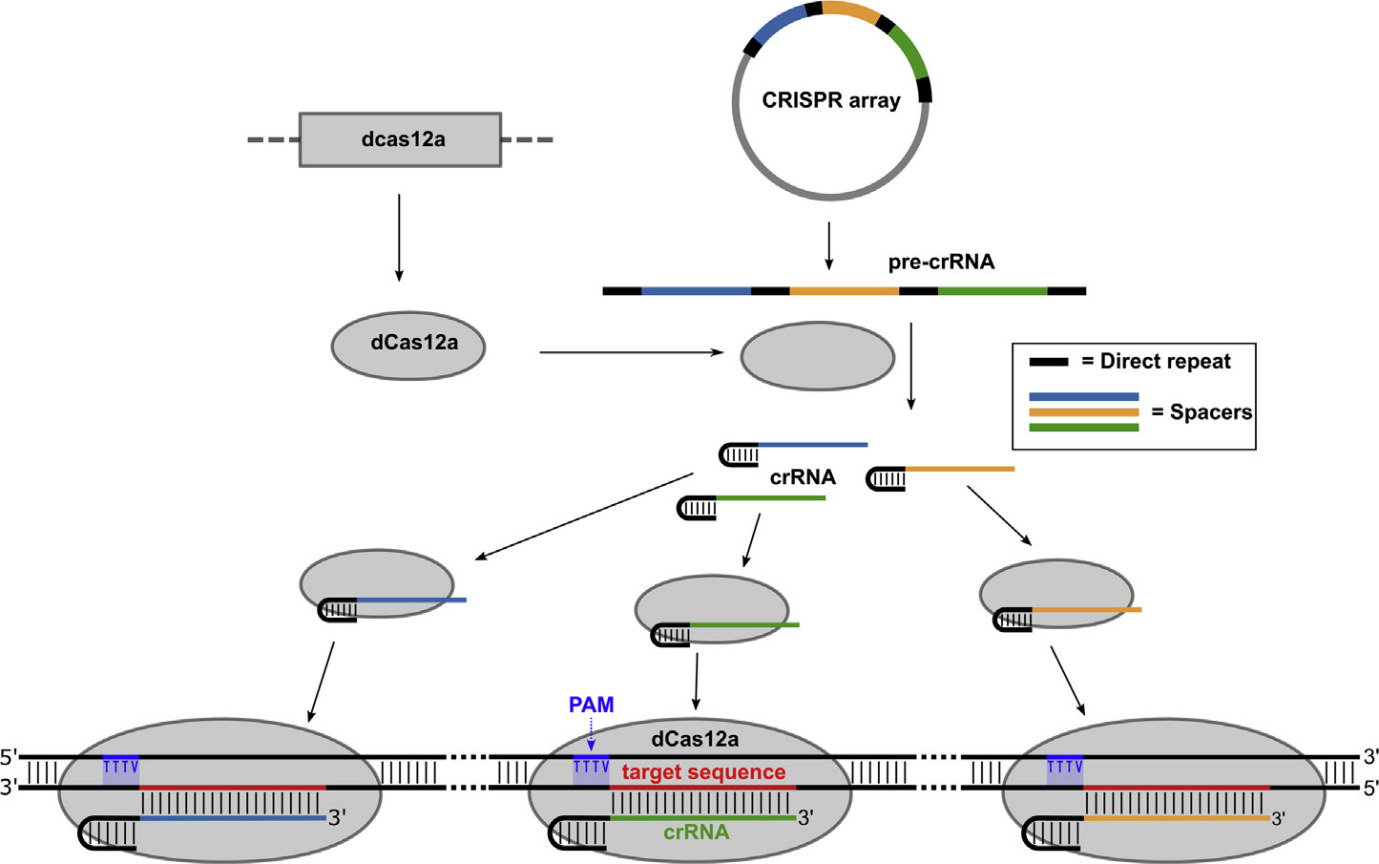
**Schematic of dCas12a-based CRISPRi.** Inducible, synthetic CRISPR arrays are transcribed into pre-crRNA and processed into mature crRNAs by dCas12a, which lacks DNA nuclease activity but retains RNA processing activity. The crRNAs direct dCas12a to the target DNA sequence(s), resulting in reduced transcription of one or multiple targets.

## Results

**Figure 2:**
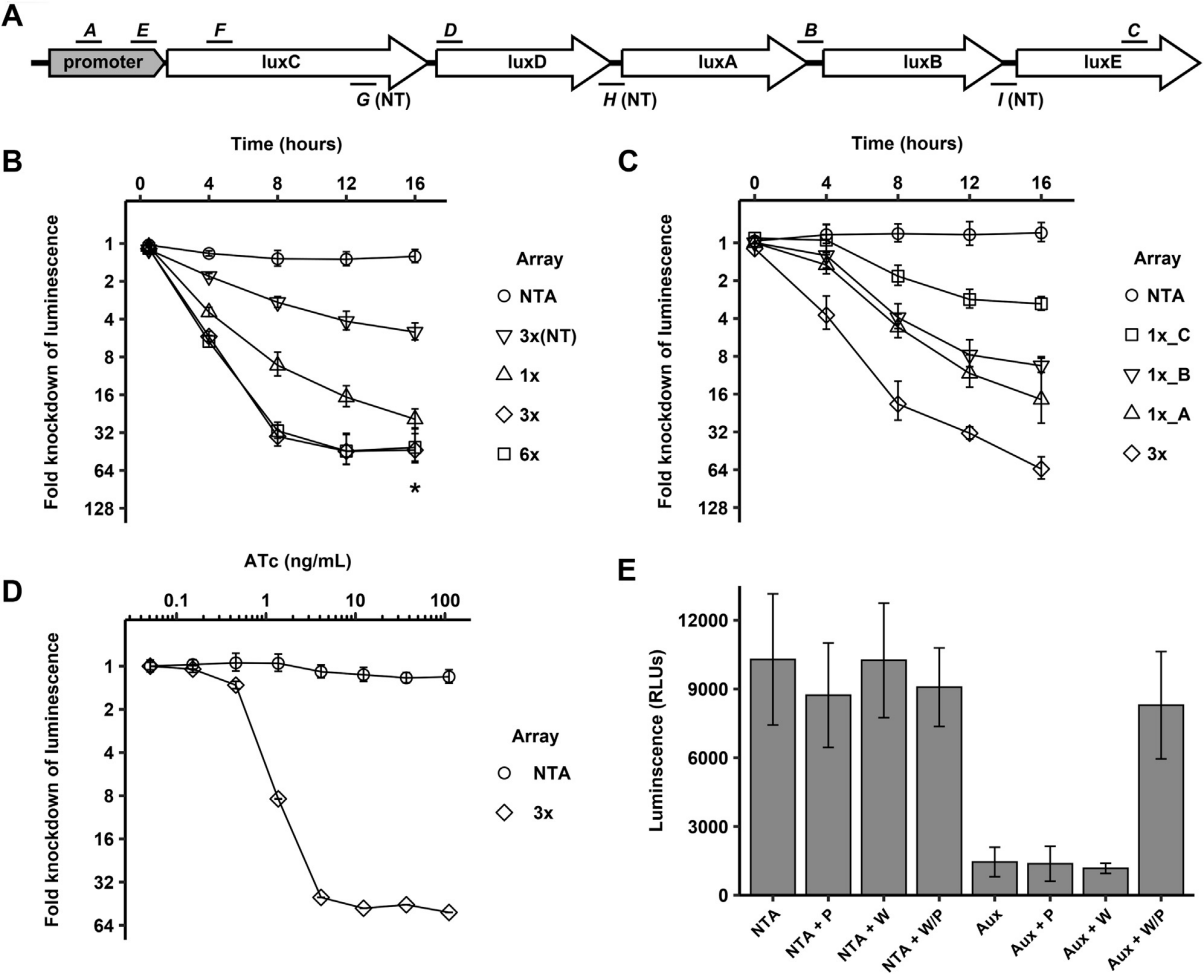
**dCas12a-based CRISPRi in *Msm*.***A*, schematic of targeting locations of the spacers used to knock down the *luxCDABE* operon. Spacers *A*, *B*, *C*, *D*, *E*, and *F* mark the six spacers used to target the template (T) strand. *G*, *H*, and *I* mark the three spacers targeting the nontemplate (NT) strand. *B*, gene repression in *Msm*. Luminescence was knocked down in *Msm-luc-dCas12a* strains by expressing synthetic CRISPR arrays with 1, 3, or 6 spacers targeting the *luxCDABE* operon or the nontargeting array. Knockdown of the *lux* operon was assessed by monitoring luciferase activity for 5 days post induction. Data are shown for each construct as the ratio of luminescence between the mock-induced samples and the ATc-induced samples for each construct. Array 1x contains spacer *A*. Array 3x contains spacers *A*, *B*, and *C*. Array 6x contains *A*, *B*, *C*, *D*, *E*, and *F*, and array 3x(NT) contains the *G*, *H*, and *I* NT spacers. NTA: Nontargeting array carrying three spacers against sequences not found in *Msm* or *Mtb*. *Asterisk* represents the assay’s limit of blank at the final timepoint. Error bars indicate standard deviation of six biological replicates. *C*, additive gene repression. Luminescence was knocked down in *Msm-luc-dCas12a* strains expressing the 3x array or its three individual spacers, 1x_A, 1x_B, and 1x_C. Error bars indicate standard deviation of three biological replicates. *D*, ATc dose–response for tuning knockdown. *Msm-luc-dCas12a* strains expressing either the 3x array or NTA were grown to early log phase and induced with ATc from 0.07 to 100 ng/ml or mock-induced with DMSO, and luminescence knockdown was measured. Data are shown as the ratio of luminescence between the mock-induced strains and the ATc-induced strains after 16 h. Error bars indicate standard deviation of three biological replicates. *E*, multigene repression in *Msm*. A synthetic array containing spacers targeting the essential Trp and Pro biosynthesis genes *trpD* and *proC* (Aux) was expressed in *Msm-luc-dCas12a*. Strains were grown in media supplemented with L-tryptophan (W) and L-proline (P), washed, diluted in media containing the indicated combinations of W and P supplementation, and growth was assessed after 12 h by measuring luminescence. Only chemical supplementation of both amino acids recovered growth, indicating double knockdown of both *trpD* and *proC* genes. Error bars indicate standard deviation of five biological replicates.

To test whether dCas12a in conjunction with a synthetic CRISPR array can be used for CRISPRi in mycobacteria, we stably introduced the gene for the inactive *Francisella novicida* Cas12a mutant Asp917Ala (dCas12a) into the Tweety recombination site of *Mycobacterium smegmatis* (*Msm*) strain mc^2^155 (19). The Asp917Ala mutant abrogates DNA cleavage activity but retains pre-CRISPR RNA processing activity (12). Initially, we could not detect the *Francisella*-derived dCas12a enzyme in *Msm* by Western blot, even after expression from a strong constitutive promoter. The *Francisella cas12a* sequence is AT-rich (30% GC), while mycobacterial genomes are GC-rich (*Mtb* 66%, *Msm* 67% GC). To test whether these differences limit expression, we tested a *Francisella cas12a* gene that was previously codon-optimized for human expression and has a GC content of 46%. This construct was readily expressed in *Msm* and *Mtb*. We next sought to test whether *Msm* expressing dCas12a can process synthetic CRISPR arrays into functional crRNAs and repress transcription of target genes. We first created the *Msm-luc-dCas12a* strain by integrating an ATc-inducible *dcas12a* into the Tweety recombination site and integrating a constitutively expressed *luxCDABE* operon, which generates autoluminescence in mycobacteria (20), into the L5 recombination site. We then targeted the template strand of the *luxCDABE* operon by episomally expressing synthetic CRISPR arrays. The CRISPR arrays contained the *Francisella* repeat sequence (GTCTAAGAACTTTAAATAATTTCTACTGTTGTAGAT) flanking each 22 bp spacer sequence complementary to the *luxCDABE* promoter and coding sequence (Fig. 2*A*). Each target region on *luxCDABE* was selected to be immediately downstream from the protospacer adjacent motif (PAM) TTTV or TTN that licenses *Francisella* Cas12a binding to the target DNA (11, 21). The synthetic CRISPR arrays for production of pre-crRNA were expressed from an inducible mycobacterial expression plasmid under the control of ATc in the *Msm-luc-dCas12a* strain. After induction, we continuously measured luciferase activity for 16 h. As controls, we included a strain expressing a nontargeting array (NTA) containing three 22 bp spacer sequences that lack homology to *Msm* or *Mtb* sequences and compared luminescence in each strain with and without ATc.

We observed reduction of luminescence in all dCas12a strains carrying arrays with lux spacers when compared with the noninduced strains and the NTA strain. Reduction in luminescence was dependent on the number of spacers targeting the operon, from 15-fold with one targeting spacer (1x) to ∼45-fold with three (3x) and six spacers (6x), indicating that three spacers may be sufficient for maximal knockdown. Maximal knockdown was reached after 8 to 12 h or 3 to 4 doubling times. To test the strand preference of dCas12a-mediated knockdown in mycobacteria, we next targeted the nontemplate strand of the lux promoter and sequences in *luxB* and *luxD* with a total of three spacers. Targeting the nontemplate strand reduced expression of lux genes, but to much lower degrees than the same number of spacers targeting the template strand (Fig. 2*B*), consistent with previous findings that the template strand is the main target for *Francisella* Cas12a (16, 21). Growth of *Msm* was marginally slowed by expression of dCas12a and the NTA (Fig. 2*B*). To test whether the use of multiple spacers targeting a single transcript has an additive effect on knockdown, we tested the 3x array compared with each of its three spacers individually (Fig. 2*C*). The individual spacers knocked down luminescence with declining efficiency in the order in which they bind to the *luxCDABE* operon, and their combined effect was in fact larger than the sum of the individual effects, suggesting moderate synergy between spacers. When we expressed dCas12a from a strong constitutive promoter, we observed baseline knockdown of about tenfold even in the absence of array induction by ATc (data not shown), indicating that even small amounts of leaky expression of the array can lead to knockdown. Strains expressing both the array and dCas12a from ATc inducible promoters allowed for a wider range of induction and were used for this study. To explore the ATc dose response of knockdown, we tested the effect of a range of ATc concentrations on luminescence (Fig. 2*D*). Small concentrations of ATc as low as 4 ng/ml fully induced maximal knockdown, and lower concentrations induced partial knockdown. These data show that dCas12 can process pre-crRNA into mature crRNAs and repress endogenous genes in *Msm*.

Although the reduction of luminescence was likely due to the simultaneous repression of several lux genes, the common final readout for all lux genes (luminescence) in these experiments could not conclusively distinguish between single or multigene knockdown. To test for functional multigene knockdown of endogenous genes, we next created a double auxotroph strain of *Msm* that carried a synthetic CRISPR array targeting two essential genes that are required for the synthesis of the amino acids Trp (*trpD*) and Pro (*proC*). We introduced two spacers for each gene into a single CRISPR array. After induction of the array in *Msm* expressing dCas12a, the strain showed no growth in medium lacking Trp and/or Pro (Fig. 2*E*). Complementation by the two amino acids fully restored growth to wild type levels, whereas complementation with either one of the two amino acids did not rescue growth (Fig. 2*E*). These data show that both genes were effectively silenced and that dCas12a can target multiple endogenous genes as well as essential genes.

**Figure 3:**
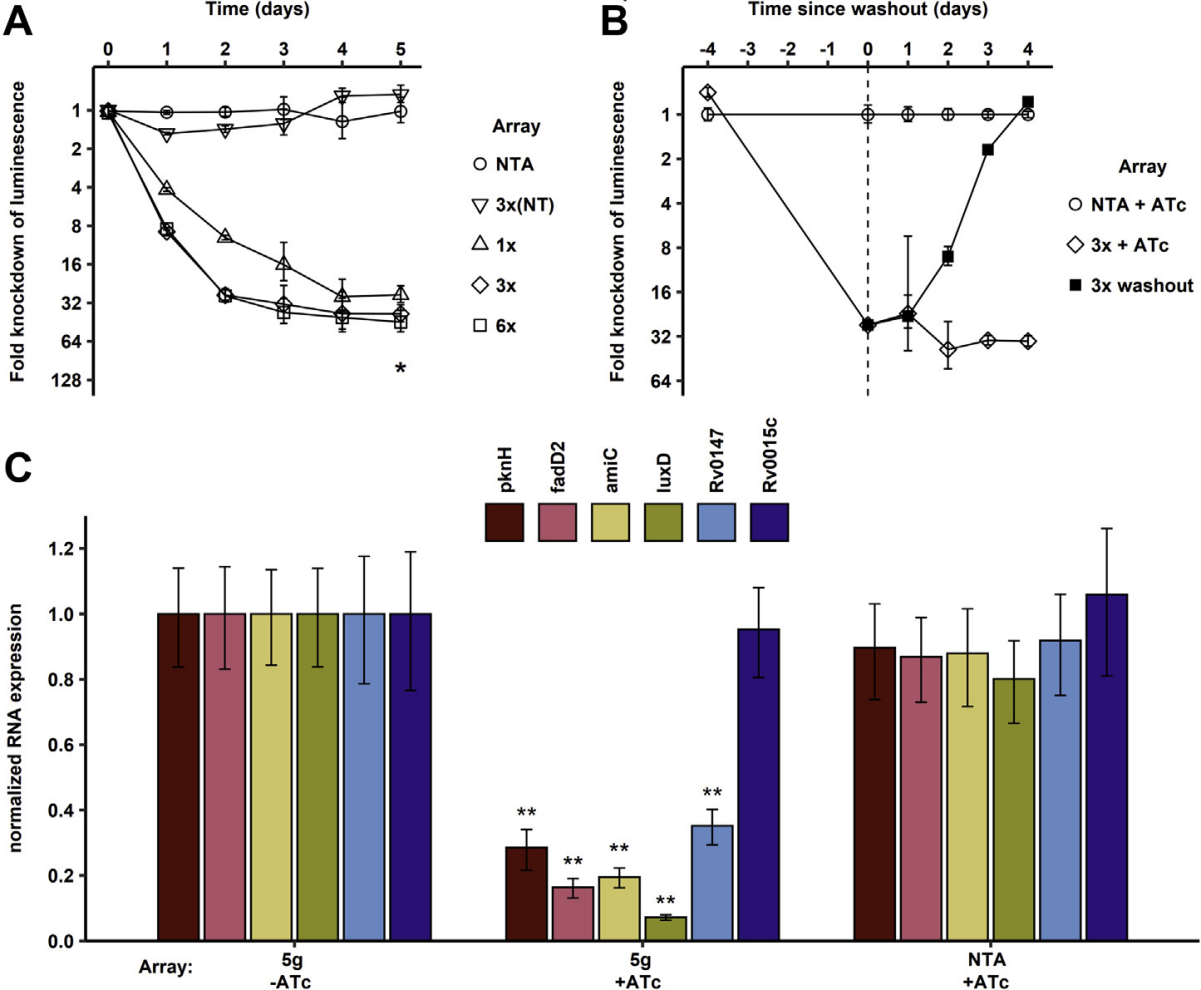
**Cas12a-based CRISPRi in *Mtb*.***A*, gene repression in *Mtb*. Luminescence was knocked down in *Mtb-luc-dCas12a* strains by expressing synthetic CRISPR arrays with 1, 3, or 6 spacers targeting the *luxCDABE* operon or the nontargeting array. Knockdown of the *lux* operon was assessed by monitoring luminescence for 5 days after induction. Data are shown as the ratio of luminescence between the mock-induced samples and the ATc-induced samples for each construct. NTA: Nontargeting array carrying three spacers against sequences not found in *Mtb*. *Asterisk* represents the assay’s limit of blank at the final timepoint. Error bars indicate standard deviation of six biological replicates. *B*, reversibility of repression. After array induction and lux repression for 4 days, ATc was washed out. Luminescence remained repressed when ATc was added back, but completely recovered after 4 days without ATc. Luminescence was normalized to that of the NTA-carrying strain. Error bars indicate standard deviation of six biological replicates. *C*, multigene knockdown in *Mtb*. Five genes were targeted in *Mtb* by a single array (5g), and knockdown was measured by qRT-PCR. Rv0015c was not targeted by the 5g array expression and served as a control. Error bars indicate standard deviation of three biological replicates. *Asterisks* indicate *p* < 0.001 compared with the mock-induced 5g strain. NTA, Nontargeting array.

We next tested whether dCas12a-mediated CRISPRi can be adapted to *Mtb*. We introduced synthetic CRISPR arrays containing spacers targeting the *luxCDABE* operon into *Mtb-luc-dCas12a*, an *Mtb* strain carrying dCas12a in the Tweety recombination site, and the lux operon in the L5 recombination site. Induction of the arrays with ATc resulted in efficient reduction of luminescence (Fig. 3*A*). The degree of knockdown was dependent on the number of spacers targeting the operon, with one spacer resulting in 28-fold and three and six spacers resulting in 45-fold reduction of luminescence signal. Similar to *Msm*, three spacers were sufficient for maximal knockdown and knockdown was maximal after 3 to 4 doubling times. In contrast to *Msm*, targeting the nontemplate strand with three spacers did not reduce the luminescence signal. Expression of dCas12a and the NTA did not have an apparent effect on growth of *Mtb* (Fig. 3*A*). To test the reversibility of the system, we induced the *Mtb* strain carrying an array with three spacers targeting the *lux* operon for 4 days and removed the inducer ATc by washout and resuspension in fresh medium. When ATc was added back to the cultures immediately after washing, the luciferase activity remained repressed. Without ATc, luciferase signal completely recovered after 4 days (Fig. 3*B*). These data show that the system is highly responsive and can be turned off within four doubling times by removing the inducer ATc. To test the selectivity of spacer binding and knockdown, we measured global gene expression in the induced and uninduced strains carrying the 6x luciferase array by RNA sequencing. While there were no significant expression changes consistent with direct off-target effects of the six spacers, a small number of genes were moderately upregulated. None of these genes had detectable similarity to the spacer sequences, and their induction may be a cellular response to the higher consumption of ATP by luciferase in the uninduced strain (Fig. S1). Next, we sought to test for multigene regulation in *Mtb*. We designed an array targeting the genes *pknH*, *fadD2*, *amiC*, *luxD*, and *rv0147* with two spacers for each gene—the 5g array. To detect knockdown of all targeted genes and determine the efficiency of multigene knockdown, we analyzed the knockdown strains by qRT-PCR. All five targeted genes were repressed, with between 3- and 14-fold reduction in mRNA (Fig. 3*C*). Transcript levels of the unrelated gene Rv0015c, which was not targeted by the array, were unchanged, indicating that expression of dCas12a and ten spacers had no general effects depressing global transcription. These data show that at least five genes can readily be repressed by dCas12a simultaneously.

**Figure 4:**
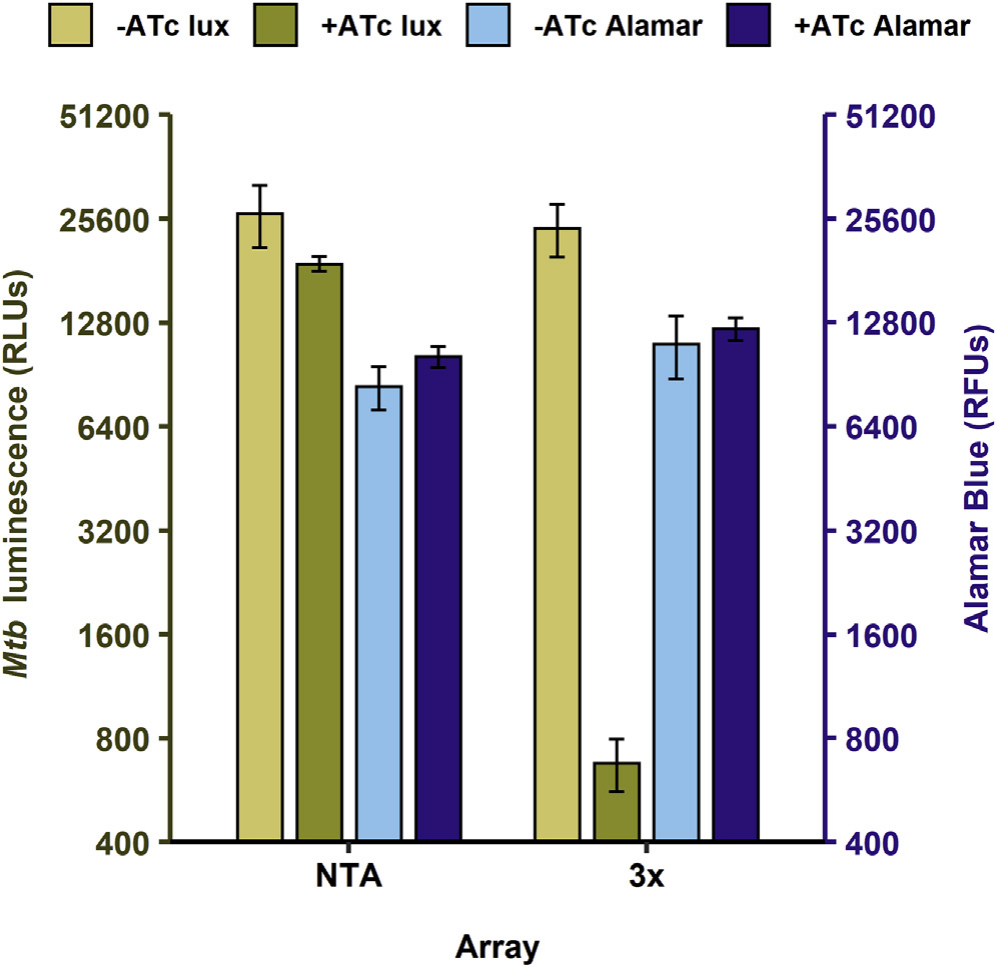
**Cas12a-based CRISPRi in an *Mtb ex vivo* infection model.** THP-1 cells were infected with an *Mtb-luc-dCas12a* strain carrying a synthetic array with three spacers targeting the *luxCDABE* operon (3x). Array expression was induced after infection and luminescence measured after 4 days. Alamar Blue reports on THP-1 fitness. The minimum bound of the left y-axis represents the limit of detection for the luminescence assay. Error bars indicate standard deviation of four replicates.

CRISPRi in mycobacteria has thus far only been used to regulate bacterial genes *in vitro* (3, 4, 5). To test whether the dCas12a-based system can also be used to regulate gene expression during infection, we infected activated human monocytic THP-1 cells, a macrophage-like cell line commonly used for *Mtb* infection (22), with a strain carrying an array with three spacers targeting the *luxCDABE* operon. CRISPRi was induced with ATc or mock-induced with DMSO after infection with *Mtb*. After 4 days, the CRISPRi strain carrying the array showed 40-fold knockdown of luminescence, comparable to the knockdown observed *in vitro* (Fig. 4). To rule out that CRISPRi led to increased THP-1 killing, which would affect *Mtb* survival and confound the luminescence readout, we measured THP-1 cell health with the Alamar Blue reagent. CRISPRi-mediated gene repression had no effect on THP-1 cell health, indicating that the reduction in luminescence was entirely due to luciferase gene repression (Fig. 4).

## Discussion

The introduction of CRISPRi to the toolkit of mycobacterial genetics has greatly accelerated many areas of mycobacterial research. Yet, the canonical Cas9-based system has some limitations, and in particular multigene knockdown remains challenging. Here, we exploited the inherent multigene targeting capability of CRISPR systems, in particular the minimal Cas12a, to achieve more efficient multigene regulation in mycobacteria. Although the upper limit for the number of genes that can be repressed with this system remains to be determined, it is likely higher than the five tested here. To knock down five genes in our study, we used ten spacers that could also be directed at different targets, although fewer spacers per gene likely affect the efficiency of any individual knockdown. The natural *Francisella* CRISPR arrays contain up to 26 spacers, perhaps indicating the natural upper limit for the system. The degree of knockdown by CRISPRi can generally be tuned by varying the concentration of the inducer ATc, by the choice of spacers, and by using target sequences with different PAM strengths. The latter two are less predictable and require empirical testing. In our system, knockdown efficiency can further be tuned by varying the number of spacers targeting each gene, a more predictable approach as each additional spacer produces higher levels of knockdown. In general, an alternative CRISPRi system provides additional options for targeting genes that may be difficult to target in other systems. For multigene knockdown, we anticipate a trade-off between the efficiency of knockdown and the number of genes targeted that should be considered for optimal array design. The repetitive nature of the repeat sequences of the synthetic arrays also introduces challenges for gene synthesis, although we could readily obtain functional arrays with as many as 13 repeats.

For facile multigene regulation, Cas12a has clear advantages over Cas9-based CRISPRi systems. The much shorter repeat and spacer sequences (∼36 bp and ∼22 bp, respectively) and the single transcriptional unit required for any number of spacers make the Cas12a-based system more practical than the current Cas9-based system, which requires crRNAs of >100 bases with individual promoter and terminator sequences. Another advantage of Cas12a-based CRISPRi is its genome coverage. The genome coverage of CRISPRi is determined by the PAM frequency for a given Cas enzyme and is a main factor for a CRISPRi system’s utility. The Cas12a PAM coverage of the *Mtb* genome is more complete than that of *S. thermophilus* Cas9, the currently most efficient Cas9 used in mycobacteria (4). The strongest PAM for *S. thermophilus* Cas9, AGAAG, is found in 34% of *Mtb* coding sequences, whereas the strongest Cas12a PAM, TTTV (V = A/C/G) is found in 89% of *Mtb* coding sequences and targets on average every 235 base pairs. Combined with less efficient PAMs for both enzymes (4, 21, 23), *S. thermophilus* Cas9 can target the *Mtb* genome every 45 bps on average for a given strand, Cas12a every 32 bp, and both can target 98% of genes. Although the *S. pyogenes* Cas9 uses a PAM that is more common, the enzyme is not as efficient in gene repression in mycobacteria (3, 4). Additional Cas12a orthologs with less stringent PAMs have recently been described (24), for example, an engineered mutant of the Cas12a enzyme from *Acidaminococcus* (25) that recognizes the PAM sequence TYCV (Y = C/T), which could further increase genome coverage. While we did not directly compare the efficiency of Cas9 and Cas12a-based CRISPRi, Cas9-based CRISPRi in *Msm* reduced luminescence by 2.7- to 166-fold in one study (4), depending on the Cas9 ortholog used, compared with maximal reduction in luminescence of 45-fold in our study. The large differences between the efficiency of Cas9 orthologs suggest that other Cas12a orthologs may also yield improved repression.

When compared to the canonical dCas9-based system, our system is particularly beneficial for dissecting redundancy between *Mtb* genes, which has impeded the study of many gene families such as the PE and PPE genes. Multidrug therapy is a bedrock of tuberculosis therapy and developing such therapies requires testing of complex gene–gene and drug–gene interactions. With our system, the testing of interactions between multiple genes to identify synergy and/or synthetic lethality is becoming tractable. The dCas12a-based CRISPRi system suppressed gene expression as efficiently in infected THP-1 cells as it did *in vitro*, which expands the system’s use to the study of genes conditionally essential during infection and to host–pathogen interactions. Because the tetracycline promoter–inducer system used here for the control of dCas12a and the synthetic arrays is also generally functional in mice, we anticipate that our system also works *in vivo*. In addition, the equal efficiency of knockdown *in vitro* and in macrophages will allow for the probing of host–pathogen interactions, for example, interactions between host-induced *Mtb* efflux pumps that currently limit the effectiveness of several tuberculosis drugs (26).

Together, we introduce a new CRISPRi system for mycobacteria for tunable and efficient multigene regulation *in vitro* and in infected macrophages. This Cas12a-based system is versatile and the efficient multigene regulation will be particularly useful for the study of larger and redundant gene families and generally for the study of higher-order genetic and host–pathogen interactions.

## Experimental procedures

### Media and growth conditions

*Mycobacterium tuberculosis* and *M. smegmatis* were grown at 37 °C in Middlebrook 7H9 broth or on 7H10 plates supplemented with 0.5% glycerol, 10% OADC, 0.05% Tween80 (broth only) with appropriate selective antibiotics and amino acids. Antibiotics were used at the following concentrations: ATc: 50 ng/ml (unless otherwise noted), Hyg: 100 ng/ml, Zeo: 25 μg/ml. L-tryptophan and L-proline were supplemented at 50 μg/ml.

### Array design and cloning

Spacer sequences were chosen by first identifying all Cas12a-compatible PAMs within a gene’s promoter and coding sequence. Target sequences containing the ideal PAM, TTTV, were prioritized over targets with the less effective PAM TTN, and target sequences within the promoter and nearest the 5′ end of the coding sequence were prioritized over downstream sequences. Off-target binding of spacers was predicted by Cas-OFFinder (27), and any spacers with potential off-target effects were omitted. The pJEBTZ integrating plasmid was constructed by combining the Tweety-phage integrating backbone of pTTP1A (11), generously provided by the Hatfull lab, with the Zeocin selectable marker from psigE, and the *E. coli* origin of replication from pJEB402-dCas10. Fn-Cpf1, with the nuclease deactivating mutation Asp917Ala and codon optimized for expression in humans, was sourced from pTE4999 (17) and inserted into the expression locus of pJEBTZ. The pJOBTZ vector was created by replacing the MOPS promoter in pJEBTZ with the TetR-regulated promoter p766 from pJR965 (4). The *Renilla* luciferase *luxCDABE* operon was expressed under the control of the constitutive MOPS promoter on an L5-integrating plasmid containing a Kan-selectable marker. The pNFCF vector was created by replacing the Uv15-Tet promoter in pDTCF (18) with the synthetic TetR-regulated promoter p766 from pJR965. To clone crRNA arrays into pNFCF, sequences flanked by overhangs for Gibson Assembly matching the vector insertion site (5′-CCGCATGCTTAATTAAGAAGGAGATATACAT-3′) – array sequence – (5′-GACTACAAGGATGACGACGACAAG-3′) were synthesized (Genscript) and obtained in a pUC57-Kan vector. The inserts were amplified using standard PrimeStarHS PCR chemistry with 55 °C annealing temp and the forward primer (5′-CCGCATGCTTAATTAAGAAGGAGATATACAT-3′) and the reverse primer (5′-CTTGTCGTCGTCATCCTTGTAGTC-3′). The pNFCF vector was PCR linearized with forward (5′-ATGTATATCTCCTTCTTAATTAAGCATGCGG-3′) and reverse (5′-GACTACAAGGATGACGACGACAAG-3′) linearization primers and a touchdown thermocycler program using 60 to 55 °C annealing temperatures. Array inserts and linearized pNFCF vector were gel-purified and then assembled *via* Gibson Assembly. All vectors used in this study are described in Table S2.

### Autoluminescence assays

Strains in log phase were grown for at least two doublings and diluted to OD_600_ of 0.005, and expression of the crRNA array and dCas12a was induced with ATc or mock-induced with DMSO. To assess knockdown, 100 μl culture was dispensed into 96-well, white, flat-bottom plates, and luminescence was quantified using a PHERAstar plate reader (BMG Labtech), blanked against media. For *M. smegmatis*, cultures were incubated inside the plate reader at 37 °C for the duration of the experiment.

### Auxotroph supplementation assay

*M. smegmatis* cultures were grown to late log-phase in media supplemented with 50 μg/ml L-tryptophan and L-proline, then diluted to an OD_600_ of 0.4 and grown for 3 h in the dark. Cultures were washed twice in nonsupplemented media and diluted to an OD_600_ of 0.1 with ATc and Hyg to drive expression of and maintain selection for the CRISPRi system. Different combinations of amino acids were supplemented into the cultures, and growth was monitored after 12 h at 37 °C by measuring luminescence.

### qRT-PCR analysis of multigene knockdown

Liquid cultures of *Mtb-luc-dCas12a* were induced in early log phase with 50 ng/ml ATc or mock-induced with DMSO and grown for 4 days to mid-log phase. RNA was extracted in Trizol, purified, and cDNA was synthesized using the SuperScriptIV polymerase and random hexamer primers. mRNA expression levels for each gene were determined by qRT-PCR using SybrGreen iTaq chemistry and normalized to sigH mRNA expression and plotted as the ratio of mRNA levels (calculated as 2^−ΔΔCq^) in each strain relative to the mock-induced 5g strain.

### RNA-seq

*Msm-luc-dCas12a* cultures were induced with ATc in triplicate and grown overnight to mid-log phase. RNA was extracted in Trizol, purified with QIAGEN RNeasy columns, and rRNA was depleted using the siTOOLs Pan-Prokaryote riboPOOL system. The cDNA library was generated using the NEBNext Ultra II RNA Library Prep Kit, and samples were sequenced on the Illumina NextSeq High Output 75 platform with paired end reads. The genomic reads were assembled and mapped using the R package DuffyTools (28, 29), the *luxCDABE* reads were mapped using SAMtools, and statistical data were generated using the EdgeR analysis within DuffyTools. Then q-values were calculated from the EdgeR *p*-values using the R package Bioconductor.

### Macrophage infection assay

Undifferentiated THP-1 cells were dispensed into a 96-well plate and differentiated with 100 nM PMA for 4 days, then infected with *Mtb-luc-dCas12a* strains at a multiplicity of infection of 1. The infection medium was replaced with fresh medium containing either 100 ng/ml of the inducer ATc or DMSO for mock induction. Knockdown was assessed by monitoring *Mtb* auto-luminescence daily *via* plate reader, and THP-1 health was visually assessed daily. After 4 days postinfection, THP-1 survival was assessed by adding Alamar Blue at 10% v/v and measuring fluorescence (560 nm excitation, 590 nm emission) after incubation for 6 h.

## Data availability

This study contains Fig. S1 and Tables S1 and S2. All original data are contained within this article.

## Supporting information

This article contains supporting information (20).

## Conflict of interest

The authors declare that they have no conflicts of interest with the contents of this article.
