# Supplementary material for: A Cas12a-based CRISPR interference system for multigene regulation in mycobacteria
Source: J Biol Chem. 2021 Jul 21;297(2):100990. doi: 10.1016/j.jbc.2021.100990 (PMC8363830; doi:10.1016/j.jbc.2021.100990)
Supplement: Supplemental Figure S1 and Tables S1–S2 [file mmc1.docx]

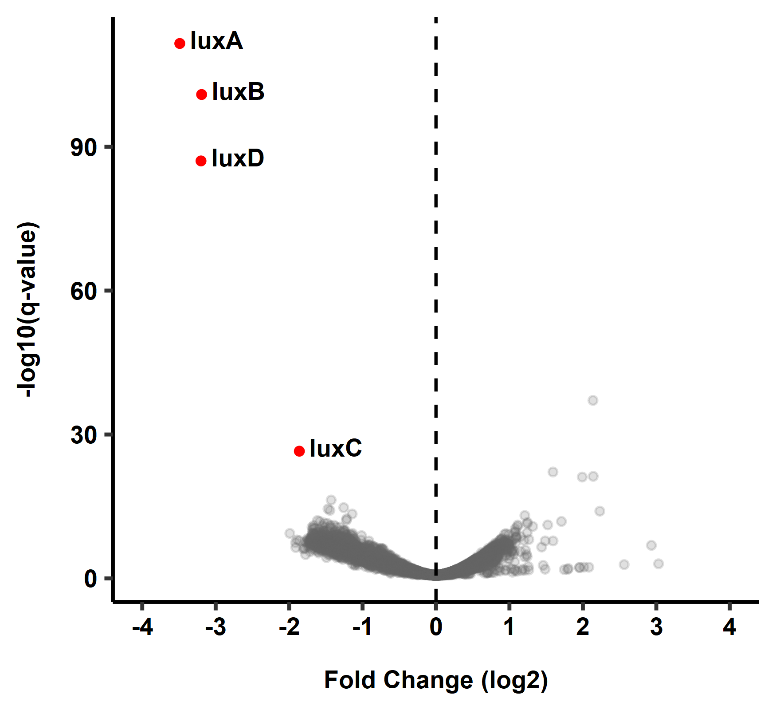
**SUPPLEMENTARY MATERIAL

Figure S1:**

**Selectivity of Cas12a-based CRISPRi.**

The gene expression changes upon CRISPRi knockdown in the *Mtb-luc-dCas12a* strain carrying a 6x array compared to uninduced.

**Table S1:** Spacer sequences

| **array/spacer** | **target** | **spacer sequence** | **PAM** |
| --- | --- | --- | --- |
| 5g_array | fadD2 | CGAACTCGACGAGGCCGCACA | TTTV |
| 5g_array | pknH | GGCCCTACCACCTCAAACGGC | TTTV |
| 5g_array | amiC | GTTGACGATGCCCTTGGTGAC | TTTV |
| 5g_array | mlux | TGCTTCCGGCTCGTATAATGTGT | TTTV |
| 5g_array | Rv0147 | TATTGCTGGTTCAACCGGGTGA | TTTV |
| 5g_array | fadD2 | TCGCACGTGCCGTTCAAGGCC | TTTV |
| 5g_array | pknH | GCGAGCGGATGAAACGCGAAG | TTTV |
| 5g_array | amiC | GACGTGGCGCGAGACGCGGC | TTTV |
| 5g_array | mlux | TCCCGGGCACTCGACAGGAGGAG | TTTV |
| 5g_array | Rv0147 | GGATGAAAAGCAAACCGATGT | TTTV |
| auxotroph | proC | GTCACCATCGCGGCCGGCGTCGG | TTTV |
| auxotroph | proC | CGGAGAAGTTCCCCGAGCGTGCC | TTN |
| auxotroph | trpD | GCGGTGTCGATGAAGATGAAGCG | TTTV |
| auxotroph | trpD | GTGAGCTCGCCGACATCATGCTC | TTN |
| mlux *A* | lux operon | TGCTTCCGGCTCGTATAATGTGT | TTTV |
| mlux *D* | lux operon | ATGGAGAACAAGTCCAAGTACAA | TTTV |
| mlux *B* | lux operon | TCCCGGGCACTCGACAGGAGGAG | TTTV |
| mlux *C* | lux operon | AGGACGAGATGCAGCGCAAGCGC | TTTV |
| mlux *E* | lux operon | GGATCCAGCTGCAGAATTCAGGA | TTN |
| mlux *F* | lux operon | CCCGAGAGCGACGACCTGGTCCA | TTN |
| mlux *G*(NT) | lux operon (non-template) | CGGGACGAAGACCAGGAACTTGT | TTTV |
| mlux *H*(NT) | lux operon (non-template) | CTCCTGTGGAGTGCAATTCTTAC | TTTV |
| mlux *I*(NT) | lux operon (non-template) | GGTCCTCCTGTGGAGTGTCTAGA | TTTV |
| NTA | non-targeting | GTCCAGGACGCCACGCGTAGTG | - |
| NTA | non-targeting | ACCAAGGACACATTCGAGCTCT | - |
| NTA | non-targeting | CAGCTCTTTCAGTATCATGGAG | - |

**Table S2:** Plasmids used in this study

| **Plasmid** | **Relevant Features** | **Antibiotic resistance** | **Reference** |
| --- | --- | --- | --- |
| pJOBTZ_hdcas12a | Plasmid for integrating ATc-inducible dcas12a (codon optimized for human) into the Tweety integration site of mycobacteria | Zeo | This study |
| pJEBTZ_hdcpf1 | Plasmid for integrating constitutively expressed dcas12a (codon optimized for humans) into the Tweety integration site of mycobacteria | Zeo | This study |
| pmlux | Plasmid for integrating constitutively expressed luxCDABE operon into mycobacterial L5 site | Kan | Andreu N, et al. (20) |
| pNFCF_Cpf1_mlux1t | ATc inducible cas12a array containing one spacer targeting luxCDABE: spacer *A* | Hyg | This study |
| pNFCF_Cpf1_mlux1t_B | ATc inducible cas12a array containing one spacer targeting luxCDABE: spacer *B* | Hyg | This study |
| pNFCF_Cpf1_mlux1t_C | ATc inducible cas12a array containing one spacer targeting luxCDABE: spacer *C* | Hyg | This study |
| pNFCF_Cpf1_mlux3t | ATc inducible cas12a array containing three spacers targeting luxCDABE: spacers *A*, *B* and *C* | Hyg | This study |
| pNFCF_Cpf1_mlux3nt | ATc inducible cas12a array containing three spacers targeting luxCDABE (non-template strand): spacers *G*, *H* and *I* | Hyg | This study |
| pNFCF_Cpf1_mlux6t | ATc inducible cas12a array containing six spacers targeting luxCDABE: spacers *A, B, C, D E* and *F* | Hyg | This study |
| pNFCF_Cpf1_NTA | ATc inducible cas12a array containing three randomly generated non-targeting spacers which have no homology to *Mtb* or *Msm* | Hyg | This study |
| pNFCF_Cpf1_PFAM0147 | ATc inducible cas12a array containing two spacers each for targeting pknH, fadD2, amiC, luxCDABE operon, and Rv0147 in *Mtb* | Hyg | This study |
| pNFCF_Cpf1_aux3 | ATc inducible cas12a array containing two spacers each for targeting proC and trpD in *Msm* | Hyg | This study |
